# Self-reported smoking status and exhaled carbon monoxide in secondary preventive follow-up after coronary heart events: Do our patients tell the truth?

**DOI:** 10.18332/tpc/191843

**Published:** 2024-09-25

**Authors:** Anete Kaldal, Serena Tonstad, Jarle Jortveit

**Affiliations:** 1 University of Oslo, Oslo, Norway; 2 Department of Research, Sorlandet Hospital Trust, Arendal, Norway; 3 Department of Endocrinology, Obesity and Preventive Medicine, Section of Preventive Cardiology, Oslo University Hospital, Oslo, Norway; 4 Department of Cardiology, Sorlandet Hospital Trust, Arendal, Norway

**Keywords:** coronary heart disease, smoking cessation, exhaled carbon monoxide

## Abstract

**INTRODUCTION:**

Smoking cessation reduces the risk of myocardial infarctions (MI) and death in patients with coronary heart disease. Smoking status is frequently assessed based on self-report. The aims of this study were to compare self-reported and objectively measured (exhaled carbon monoxide [eCO]) smoking status after MI, percutaneous coronary intervention (PCI), and coronary artery bypass grafting (CABG), and to assess whether assumed wrongly declared smoking cessation was associated to poorer achievement of other treatment targets for secondary prevention.

**METHODS:**

This study was a sub-analysis from a randomized controlled trial at Sorlandet Hospital, Arendal, Norway, 2007–2022, including patients hospitalized due to MI or after scheduled PCI/CABG, and primarily aimed at comparing secondary preventive follow-up in the outpatient clinic versus primary healthcare. Participants were followed up after the index event through outpatient consultations. Smoking status was assessed by self-report and by eCO (Smokerlyzer, Bedfont, UK) with concentration values ≥6 ppm interpreted as suggesting smoking.

**RESULTS:**

A total of 1540 participants aged 18–80 years were included in the main study. Self-reported smoking status and concomitant eCO measurement one year after the index event were available in 1291 (84%) participants. In all, 358 (28%) participants reported smoking at the index event, and after one year, of which 127 (35%) of these reported that they quit smoking. The concentration of eCO was ≥6 ppm one year after the index event in 285 (22%) patients, and 72 (25%) of these patients reported non-smoking. Fewer patients with elevated eCO reporting nonsmoking achieved the treatment target for blood pressure (<140/90 mmHg) in comparison to those reporting smoking (53% vs 68%, p=0.02). No differences for the other treatment targets for secondary prevention were found.

**CONCLUSIONS:**

The study indicates a need for objective measures for smoking cessation both in clinical studies and in clinical practice, and may indicate a lack of truthfulness regarding smoking habits.

**CLINICAL TRIAL REGISTRATION:**

The study is registered on the official website of ClinicalTrials.gov

**IDENTIFIER:**

ID NCT00679237

## INTRODUCTION

Coronary heart disease (CHD) is a common cause of morbidity and mortality in Europe^1^. Although the European Society of Cardiology guidelines for secondary prevention of CHD are easily accessible and provide detailed recommendations, many patients still experience recurrent cardiovascular events^2,3^. Smoking is one of the major risk factors for CHD, and smoking cessation is potentially the most effective of all preventive measures, with substantial reductions in (recurrent) myocardial infarctions (MI) and death^4^. Quitting smoking is strongly recommended in the guidelines^2^. Diagnosis and/or treatment of CHD may be an important impetus for smoking cessation. Evidence-based guidelines recommended interventions for smoking cessation in secondary preventive follow-up after CHD events include: 1) advising the patient to stop smoking; 2) reiterating the benefits of quitting; and 3) agreeing on a specific plan with a follow-up arrangement^2^. Pharmaceutical support for stopping smoking should be considered in all smokers. However, persistent or re-uptake of smoking is common in patients with CHD^5^.

Smoking status is frequently assessed by self-report. Some patients falsely declare themselves to be non-smokers^6,7^. This can lead to an overestimation of smoking cessation rates. Objective measures of tobacco smoking may also be useful in improving clinical management and counseling of patients with difficulties quitting smoking.

The primary aim of this sub-analysis from a randomized controlled trial of hospital-based versus primary care-based follow-up after MI, percutaneous coronary intervention (PCI), and coronary artery bypass grafting (CABG), was to compare self-reported and objective measured [carbon monoxide (eCO)] smoking status. Second, we assessed the achievement of the other treatment targets for secondary prevention after MI, PCI, or CABG in patients with elevated eCO reporting smoking cessation compared to patients reporting smoking.

## METHODS

### Study design and study population

The data for this sub-analysis were retrieved from an open randomized controlled trial at Sorlandet Hospital, Arendal, Norway, in the period 2007–2022. The focus of the main study was to determine whether all-cause mortality and the composite endpoint of all-cause mortality, recurrence of non-fatal MI, new PCI/CABG, and non-fatal stroke differed between patients who received cardiovascular secondary preventive follow-up at the hospital and those within primary health care^8^. Patients who were admitted due to MI or scheduled PCI/CABG aged 18–80 years were randomized to follow-up at the hospital or within the primary health care. Lack of ability to cooperate, known alcohol or drug abuse, use of narcotics, pregnancy or breast-feeding, serious comorbidity with a life expectancy of <2 years, or participation in other secondary prevention studies were considered as exclusion criteria. Patients with a follow-up of <12 months were excluded from the analysis.

### Intervention

Patients in the intervention group were followed up by trained nurses who consulted cardiologists if necessary. For this group, the first consultation was under hospitalization for the index event, followed by outpatient consultations after the discharge^8^. Patients were consulted regarding lifestyle measures and achievement of treatment targets evaluated at each consultation. Patients were asked to report smoking status, physical activity, diet, and use of medication. Blood pressure, body mass index, waist circumference, cardiac troponin T, lipid profile, HbA1c, and eCO were assessed^8^. Smokers were strongly advised to quit smoking during index hospitalization and at all outpatient visits. Nicotine replacement therapy (NRT) was offered during hospital admission, and continuation of NRT or a 12-week course of varenicline after discharge was advised^8^.

Patients randomized to the primary care group were followed up by family physicians with respect to the cardiovascular secondary preventive measures. The treatment targets for cardiovascular secondary prevention were described in discharge papers and sent to the patient’s family physician. Patients in this group had their outpatient appointments for data collection regularly after the discharge for index events, without interfering with follow-up visits with the family physician.

### Treatment targets of secondary prevention

The secondary preventive treatment targets adhered to the latest ESC guidelines available and were as follows: no smoking, blood pressure <140/90 mmHg, LDL-cholesterol <1.8 mmol/L (<2.5 mmol/L until 2017, <1.4 mmol/L from 2020), body mass index (BMI) <25 kg/m^2^, daily use of lipid-lowering therapy, daily use of acetylsalicylic acid, and physical activity of moderate intensity ≥150 min weekly^9-14^.

### Exhaled carbon monoxide (eCO)

The eCO concentration (ppm) was measured at each study visit with a CO tester (Smokerlyzer, Bedfont, UK) and rounded to the nearest integer, with values 0–5 ppm interpreted as no smoking and ≥6 ppm suggestive of smoking^15^. For eCO measurement, subjects were asked to hold their breath for 15 s and then exhale smoothly into the disposable mouthpiece of the monitor. The device was calibrated annually. The manufacturer reports ±2 ppm (5%) accuracy and a range of 0–150 ppm^16^.

### Outcomes

The primary endpoint was self-reported smoking status in patients with eCO ≥6 ppm one year after the index coronary heart event (MI or scheduled CABG/PCI). The secondary endpoint was the achievement of treatment targets of secondary prevention for cardiovascular risk factors, and medication use one year after the index coronary heart event for patients with eCO ≥6 ppm and reporting non-smoking versus smoking.

### Statistical analysis

Continuous variables are reported as mean ± SD (standard deviation), and differences between groups analyzed using independent samples t-tests. Categorical variables are presented as frequencies and percentages, while between-group differences are analyzed by the chi-squared test. Missing values are reported, and categorical variables are reported as the proportion of non-missing values. Complete-case analysis was used to manage missing data. A p<0.05 was regarded as statistically significant. The statistical analyses were performed using STATA, version 17 (StataCorp, 4905 Lakeway Dr, College Station, TX 77845, USA).

Ethics The study was approved by Regional Committee for Medical and Health Research Ethics (REK, 2.2007.248). All study participants signed a written consent form, and principles of Declaration of Helsinki were respected. The data collection, storage and processing were approved by Norwegian Social Science Data Services (NSD, 2007/17221). The study is registered at ClinicalTrials.gov (16.05.2008, NCT00679237).

## RESULTS

**Figure 1:**
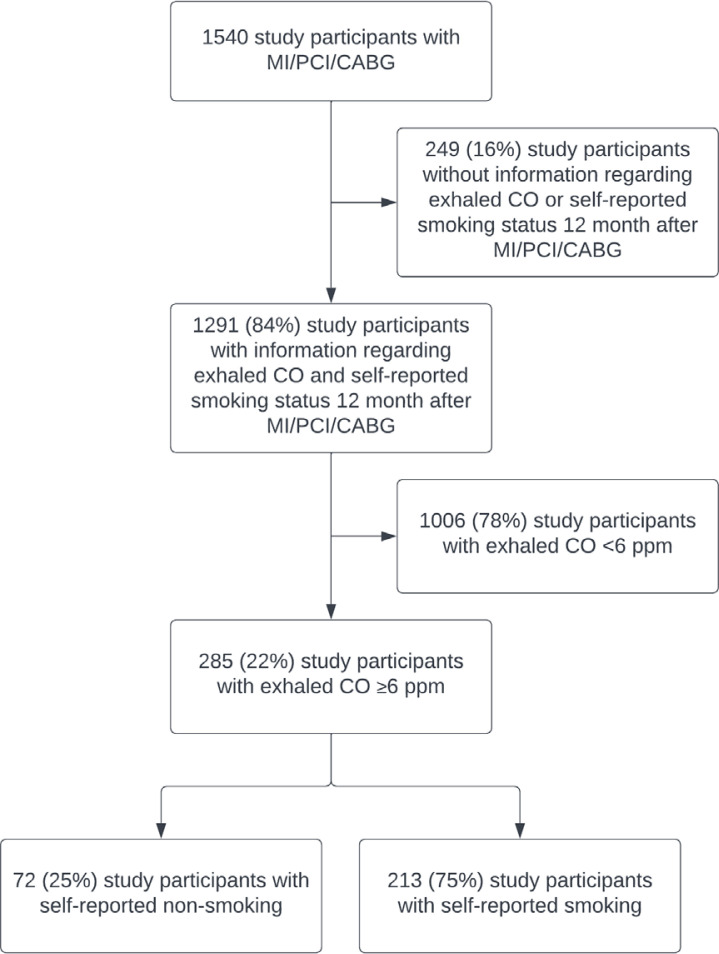
Study flow chart of participants included in a sub-analysis of self-report smoking habits from a randomized controlled secondary preventive trial after myocardial infarction (MI) or after scheduled percutaneous coronary intervention (PCI) or coronary artery bypass grafting (CABG) at Sorlandet Hospital Arendal, Norway, 2007–2022

A total of 1540 patients were included in the main study during the inclusion period from 2007 to 2017 (Figure 1). Patients with missing information regarding eCO or self-reported smoking status one year after the index event (n=249; 16%) were excluded from further analysis. Among the remaining 1291 patients, 358 (28%) patients reported current smoking, and 511 (40%) patients reported previously smoking at the index event (MI, PCI, or CABG). After one year, 127 (35%) initial smokers reported smoking cessation. A total of 258 (20%) patients reported current smoking.

### Elevated exhaled CO and self-reported smoking status

**Figure 2:**
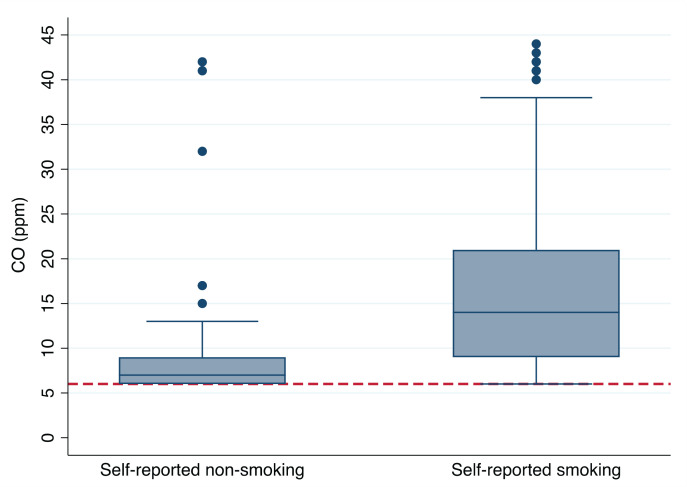
Exhaled carbon monoxide (eCO) concentration in patients self-reported smoking versus non-smoking in a sub-analysis from a randomized controlled secondary preventive trial at Sorlandet Hospital, Arendal, Norway, 2007–2022, with 1540 patients hospitalized due to myocardial infarction (MI) or after scheduled percutaneous coronary intervention (PCI) or coronary artery bypass grafting (CABG)

The concentration of eCO was ≥6 ppm one year after the index event in 285 (22%) patients, with a median value of 11 ppm (interquartile range, IQR: 7–20). A total of 72 (25%) of the patients with eCO ≥6 ppm reported non-smoking. This group constituted 7% of the participants reporting non-smoking. Among participants with eCO ≥6 ppm, median values were 7 ppm (IQR: 6–9) and 15 ppm (IQR: 9–22) in patients reporting non-smoking and smoking, respectively (Figure 2). A total of 113 (32%) initial smokers reported smoking cessation and had eCO <6 ppm one year after the index coronary heart event.

### Baseline characteristics

Baseline characteristics at the index events (MI, PCI, or CABG) of patients with eCO ≥6 ppm reporting non-smoking and patients with eCO ≥6 ppm reporting smoking one year after the index event are described in Table 1.

**Table 1:** Baseline clinical characteristics at hospitalization in patients with exhaled carbon monoxide concentration (CO) ≥6 ppm reporting non-smoking and patients with exhaled CO ≥6 ppm reporting smoking one year after myocardial infarction (MI), scheduled percutaneous coronary intervention (PCI) or coronary artery bypass grafting (CABG), Sorlandet Hospital, Arendal, Norway, 2007–2022

*Characteristics*	*Missing values*	*Self-reported non-smoking*	*Self-reported smoking*	*p^c^*
	*(N=285)* *n (%)*	*(N=72)* *n (%)*	*(N=213)* *n (%)*	
**Demographic parameters**				
Age (years), mean (SD)	0 (0)	63 (10)	59 (10)	0.002
Men	0 (0)	60 (83)	161 (76)	0.17
Married/cohabiting	3 (1)	55 (76)	144 (69)	0.21
Higher education^a^	21 (7)	20 (30)	40 (20)	0.11
Working^b^	0 (0)	32 (46)	89 (42)	0.58
**Smoking history**				
Self-reported previous smoking	0 (0)	38 (53)	17 (8)	<0.001
Self-reported smoking at baseline	0 (0)	14 (19)	194 (91)	<0.001
Cigarettes/week, mean (SD)	122 (43)	69 (43)	111 (56)	0.03
**Pharmaceutical therapy**				
Lipid lowering therapy	14 (5)	32 (47)	89 (44)	0.64
Antihypertensive therapy	8 (3)	28 (40)	87 (42)	0.77
**Medical history/comorbidity**				
BMI (kg/m^2^), mean (SD)	34 (12)	28 (4)	28 (5)	0.85
Diabetes	1 (0)	15 (21)	36 (17)	0.46
Previous myocardial infarction	3 (1)	7 (9)	33 (16)	0.23
Previous percutaneous coronary intervention	1 (0)	12 (17)	34 (16)	0.90
Previous coronary artery bypass grafting	1 (0)	2 (3)	8 (4)	0.69
Previous stroke	2 (0)	6 (8)	12 (6)	0.43
Renal failure	3 (1)	1 (1)	5 (2)	0.62
Acute myocardial infarction as index event	4 (1)	43 (61)	87 (41)	0.005
**Randomization result**				
Hospital based follow-up	0 (0)	36 (50)	106 (50)	0.97

a College and/or university education.

b Engaged in paid employment.

c p-value of independent samples t-tests for continuous variables and chi-squared tests for categorical variables.

Participants reporting non-smoking despite having an eCO ≥6 ppm were older than the patients reporting smoking. Most patients with elevated eCO reporting non-smoking one year after index event were prior smokers (n=52; 72%). Half of the patients were offered hospital-based follow-up. Greater proportion of self-reported non-smokers presented with MI as a qualifying index event.

### Achievement of treatment targets for secondary prevention

Fewer patients with elevated eCO reporting non-smoking achieved the treatment target for blood pressure [38 (53%) vs 144 (68%), p=0.02]. We found no other differences in the achievement of the targets for secondary prevention between patients with elevated eCO reporting non-smoking versus those reporting smoking (Table 2).

**Table 2:** Secondary preventive target achievement for cardiovascular risk factors and medication use in patients with exhaled carbon monoxide (CO) ≥6 ppm reporting non-smoking and patients with exhaled CO ≥6 ppm reporting smoking one year after myocardial infarction (MI), scheduled percutaneous coronary intervention (PCI) or coronary artery bypass grafting (CABG), Sorlandet Hospital, Arendal, Norway, 2007–2022

*Target achievement^a^*	*Missing values*	*Self-reported non-smoking*	*Self-reported smoking*	*p^b^*
	*(N=285)*	*(N=72)*	*(N=213)*	
	*n (%)*	*n (%)*	*n (%)*	
Blood pressure	0 (0)	38 (53)	144 (68)	0.02
LDL-cholesterol	8 (3)	58 (81)	141 (69)	0.06
Body mass index	2 (1)	20 (28)	58 (27)	0.96
Lipid lowering therapy	5 (2)	69 (99)	206 (98)	0.79
Acetylsalicylic acid	1 (0)	65 (90)	204 (96)	0.05
Physical activity	0 (0)	41 (57)	103 (48)	0.21

a Blood pressure <140/90 mmHg, LDL-cholesterol <2.5 mmol/L (2007–2017), <1.8 mmol/L (2018–2020), <1.4 mmol/L (2021–), body mass index <25 kg/m^2^, daily use of lipid lowering therapy, daily use of acetylsalicylic acid, physical activity of minimum moderate intensity ≥150 min weekly.

b p of chi-squared tests.

## DISCUSSION

In this sub-analysis from a randomized controlled secondary preventive trial of patients hospitalized due to MI or after scheduled PCI/CABG, an elevated eCO was found in 1 in 5 participants one year after the index event. A quarter of these patients reported non-smoking.

Registration of smoking habits in clinical studies may be based on self-report. Self-assessment of smoking status may lead to inaccurate measures of smoking exposure due to smoking denial or difficulty in recalling the quantity and duration of smoking. This misclassification potentially leads to an overestimation of the effect of smoking intervention and an underestimation of the biological effects of smoking cessation. Veit et al.^7^ have described a particularly high number of false negative self-reports of tobacco use among lung transplant candidates. This might be explained by the patient’s awareness of the potentially life-threatening consequences, including exclusion from transplantation. Similarly, Middleton and Morice^17^ described false negative self-reports among those with eCO >6 ppm. However, false declaration of non-smoking has no direct or immediate negative or positive consequences in secondary preventive follow-up after a CHD event. The reasons why some patients probably lie about their tobacco use are unclear. Embarrassment at not being able to stop smoking can be one of several possible explanations.

Higher age among the patients reporting non-smoking despite elevated eCO may possibly indicate age itself as a factor influencing eCO levels. Adjustment of a cut-off value according to the age group, with a cut-off value of 4 ppm and 5 ppm for those aged 16–25 years and 26–70 years, respectively, has been suggested^18^. In case higher age contributes to elevated eCO levels, it could probably explain lower median eCO levels among these patients compared to those who reported smoking, as well as contribute to higher blood pressure in this group and more advanced coronary artery disease resulting in greater proportion patients presenting with MI as an index event. The association of age and eCO might be a subject for further studies.

Except for blood pressure targets, we found no differences in the achievement of treatment targets for secondary prevention in patients with elevated eCO reporting non-smoking versus patients reporting smoking. A lower proportion of participants who achieved blood pressure treatment targets among self-reported non-smokers with elevated eCO might also support results recently described by Bradley et al.^19^, where higher eCO was associated with poorer cardiovascular health both for smokers and non-smokers. Although smoking markedly increases the risk of atherosclerotic cardiovascular events, data are inconsistent regarding the impact of smoking on the incidence of hypertension^20-22^.

Air pollution in large cities has been shown to increase dramatically eCO levels^23^. We assume this as a less possible reason for elevated eCO among self-reported non-smokers in our study, as there are no large cities within the geographical area covered.

In our opinion, this study indicates the potential usefulness of measuring eCO in secondary preventive follow-up after MI, PCI and/or CABG. Measurements of eCO may identify patients at high risk who benefit from optimalization of secondary preventive therapy, including pharmaceutic support for smoking cessation.

### Limitations

The present study has certain limitations. We did not obtain patient histories regarding passive smoking, the last cigarette smoked, smoking patterns, or the presence of chronic obstructive pulmonary disease or asthma. Those factors can influence eCO levels and could decrease the sensitivity and specificity of CO monitoring. In addition, we did not collect demographic data related to ethnic or racial characteristics. The concentration of exhaled CO reflects smoking exposure over a limited time interval and may not provide accurate estimates if there is a break in smoking. The manufacturer reports ±2 ppm (5%) accuracy, which might contribute to the incorrect classification of non-smokers who have borderline (near 6 ppm) eCO values. Statistical analyses were not adjusted for multiple testing. Furthermore, the study was limited to one hospital and a limited number of participants.

## CONCLUSIONS

Self-reported smoking status must be interpreted with caution. Some patients appear not to report their smoking status correctly. We did not delve into the reasons for this, though embarrassment or fear may be important. While not all studies of smoking cessation may be able to include objective measures of cessation, interpretation of the results of such studies will require taking the limitations of self-report into consideration. This study underlines a need for objective measures for smoking cessation both in clinical studies and in clinical practice.

## Data Availability

The study protocol and data are available on request due to privacy/ethical restrictions, so the corresponding author should be contacted if needed.

## References

[1] Timmis A, Vardas P, Townsend N, et al. European Society of Cardiology: cardiovascular disease statistics 2021. Eur Heart J. 2022;43(8):716-799. doi:10.1093/eurheartj/ehab892 35016208

[2] Visseren FLJ, Mach F, Smulders YM, et al. 2021 ESC Guidelines on cardiovascular disease prevention in clinical practice. Eur Heart J 2021; 42: 3227-3337. 2021/08/31. doi:10.1093/eurheartj/ehab484 34458905

[3] Jortveit J, Halvorsen S, Kaldal A, Pripp AH, Govatsmark RES, Langørgen J. Unsatisfactory risk factor control and high rate of new cardiovascular events in patients with myocardial infarction and prior coronary artery disease. BMC Cardiovasc Disord. 2019;19(1):71. doi:10.1186/s12872-019-1062-y 30922234 PMC6437860

[4] GBD 2017 Risk Factor Collaborators. Global, regional, and national comparative risk assessment of 84 behavioural, environmental and occupational, and metabolic risks or clusters of risks for 195 countries and territories, 1990-2017: a systematic analysis for the Global Burden of Disease Study 2017. Lancet. 2018;392(10159):1923-1994. doi:10.1016/S0140-6736(18)32225-6 30496105 PMC6227755

[5] Prugger C, Wellmann J, Heidrich J, et al. Readiness for smoking cessation in coronary heart disease patients across Europe: Results from the EUROASPIRE III survey. Eur J Prev Cardiol. 2015;22(9):1212-1219. doi:10.1177/2047487314564728 25516535

[6] Stelmach R, Fernandes FL, Carvalho-Pinto RM, et al. Comparison between objective measures of smoking and self-reported smoking status in patients with asthma or COPD: are our patients telling us the truth?. J Bras Pneumol. 2015;41(2):124-132. doi:10.1590/S1806-37132015000004526 25972966 PMC4428849

[7] Veit T, Munker D, Leuschner G, et al. High prevalence of falsely declaring nicotine abstinence in lung transplant candidates. PLoS One. 2020;15(6):e0234808. doi:10.1371/journal.pone.0234808 32555678 PMC7302701

[8] Kaldal A, Tonstad S, Jortveit J. Long-term hospital-based secondary prevention of coronary artery disease: a randomized controlled trial. BMC Cardiovasc Disord. 2021;21(1):600. doi:10.1186/s12872-021-02426-3 34915839 PMC8679993

[9] Graham I, Atar D, Borch-Johnsen K, et al. European guidelines on cardiovascular disease prevention in clinical practice: executive summary. Fourth Joint Task Force of the European Society of Cardiology and other societies on cardiovascular disease prevention in clinical practice (constituted by representatives of nine societies and by invited experts). Eur J Cardiovasc Prev Rehabil. 2007;14 Suppl 2:E1-E40. doi:10.1097/01.hjr.0000277984.31558.c4 17726406

[10] Perk J, De Backer G, Gohlke H, et al. European Guidelines on cardiovascular disease prevention in clinical practice (version 2012). The Fifth Joint Task Force of the European Society of Cardiology and Other Societies on Cardiovascular Disease Prevention in Clinical Practice (constituted by representatives of nine societies and by invited experts). Eur Heart J. 2012;33(13):1635-1701. doi:10.1093/eurheartj/ehs092 22555213

[11] Piepoli MF, Hoes AW, Agewall S, et al. 2016 European Guidelines on cardiovascular disease prevention in clinical practice: The Sixth Joint Task Force of the European Society of Cardiology and Other Societies on Cardiovascular Disease Prevention in Clinical Practice (constituted by representatives of 10 societies and by invited experts)Developed with the special contribution of the European Association for Cardiovascular Prevention & Rehabilitation (EACPR). Eur Heart J. 2016;37(29):2315-2381. doi:10.1093/eurheartj/ehw106 27222591 PMC4986030

[12] Ibanez B, James S, Agewall S, et al. 2017 ESC Guidelines for the management of acute myocardial infarction in patients presenting with ST-segment elevation: The Task Force for the management of acute myocardial infarction in patients presenting with ST-segment elevation of the European Society of Cardiology (ESC). Eur Heart J. 2018;39(2):119-177. doi:10.1093/eurheartj/ehx393 28886621

[13] Mach F, Baigent C, Catapano AL, et al. 2019 ESC/EAS Guidelines for the management of dyslipidaemias: lipid modification to reduce cardiovascular risk: The Task Force for the management of dyslipidaemias of the European Society of Cardiology (ESC) and European Atherosclerosis Society (EAS). European Heart Journal 2019; 41: 111-188. doi:10.1093/eurheartj/ehz455 31504418

[14] Collet J-P, Thiele H, Barbato E, et al. 2020 ESC Guidelines for the management of acute coronary syndromes in patients presenting without persistent ST-segment elevation: The Task Force for the management of acute coronary syndromes in patients presenting without persistent ST-segment elevation of the European Society of Cardiology (ESC). European Heart Journal 2020. doi:10.1093/eurheartj/ehaa575 33085966

[15] Benowitz NL, Bernert JT, Foulds J, et al. Biochemical Verification of Tobacco Use and Abstinence: 2019 Update. Nicotine Tob Res 2020; 22: 1086-1097. 2019/10/02. DOI: 10.1093/ntr/ntz132 31570931 PMC7882145

[16] Bedfont Scientific Ltd. PiCo Smokerlyzer Technical Specification. Accessed June 24, 2024. https://resources.bedfont.com/wp-content/uploads/2023/12/MKT377-Multilyzer-piCO-flyer-Issue-12.pdf

[17] Middleton ET, Morice AH. Breath carbon monoxide as an indication of smoking habit. Chest. 2000;117: 758-763. 2000/03/14. DOI: 10.1378/chest.117.3.758 10713003

[18] Chatrchaiwiwatana S and Ratanasiri A. EXHALED CARBON MONOXIDE LEVELS AMONG TOBACCO SMOKERS BY AGE. Southeast Asian J Trop Med Public Health. 2017; 48: 429-437 29642305

[19] Tun B, Ehrbar R, Short M, Cheng S, Vasan RS, Xanthakis V. Association of Exhaled Carbon Monoxide With Ideal Cardiovascular Health, Circulating Biomarkers, and Incidence of Heart Failure in the Framingham Offspring Study. J Am Heart Assoc. 2020;9(21):e016762. doi:10.1161/JAHA.120.016762 33100134 PMC7763395

[20] Linneberg A, Jacobsen RK, Skaaby T, et al. Effect of Smoking on Blood Pressure and Resting Heart Rate: A Mendelian Randomization Meta-Analysis in the CARTA Consortium. Circ Cardiovasc Genet. 2015;8(6):832-841. doi:10.1161/CIRCGENETICS.115.001225 26538566 PMC4684098

[21] Åsvold BO, Bjørngaard JH, Carslake D, et al. Causal associations of tobacco smoking with cardiovascular risk factors: a Mendelian randomization analysis of the HUNT Study in Norway. Int J Epidemiol. 2014;43(5):1458-1470. doi:10.1093/ije/dyu113 24867305

[22] Li G, Wang H, Wang K, et al. The association between smoking and blood pressure in men: a cross-sectional study. BMC Public Health. 2017;17(1):797. Published 2017 Oct 10. doi:10.1186/s12889-017-4802-x 29017534 PMC5634904

[23] Maga M, Janik MK, Wachsmann A, et al. Influence of air pollution on exhaled carbon monoxide levels in smokers and non-smokers. A prospective cross-sectional study. Environ Res. 2017;152:496-502. doi:10.1016/j.envres.2016.09.004 27712837

