# Erratum: Self-reported smoking status and exhaled carbon monoxide in secondary preventive follow-up after coronary heart events: Do our patients tell the truth?

**DOI:** 10.18332/tpc/193831

**Published:** 2024-11-05

**Authors:** European Publishing Production Team

**Affiliations:** 1 European Publishing, Heraklion, Greece

**Keywords:** coronary heart disease, smoking cessation, exhaled carbon monoxide

Erratum on:

Self-reported smoking status and exhaled carbon monoxide in secondary preventive follow-up after coronary heart events: Do our patients tell the truth?

By Anete Kaldal, Serena Tonstad, Jarle Jortveit

Tobacco Prevention & Cessation, Volume 10, Issue September, Pages 1–8

Publish date: 25 September 2024

DOI: https://doi.org/10.18332/tpc/191843

In the abstract of the originally published version of the article, in the “Results” section, 3rd line, the phrase “In all, Brussels, Belgium, from the 12th to the 13th of September 2024.” has been replaced by “In all, 358 (28%) participants reported smoking at the index event, and after one year, of which 127 (35%) of these reported that they quit smoking.” The mentioned change has been corrected also online.

